# Animal-to-Human SARS-associated Coronavirus Transmission?

**DOI:** 10.3201/eid1005.040022

**Published:** 2004-05

**Authors:** Zhao-Rong Lun, Liang-Hu Qu

**Affiliations:** * Zhongshan (Sun Yat-Sen) University, Guangzhou, People’s Republic of China

**Keywords:** SARS, transmission

**To the Editor**: Martina et al. reported that domestic cats and ferrets are susceptible to infection by severe acute respiratory syndrome (SARS)–associated coronavirus (SARS-CoV) isolated from a patient infected with SARS. These infected animals could efficiently transmit the virus to uninfected animals housed with them (*1*). This finding is similar to that of SARS transmission in humans in which SARS-CoV can be quickly spread from person to person through close contact. Ferrets and domestic cats not only can be infected by SARS-CoV in the laboratory, but also can shed SARS-CoV from the pharynx at 2 days postinfection and continuing through 10 and 14 days postinfection, respectively (*1*). No clinical signs were observed in six cats that were injected with SARS-CoV, whereas three of six ferrets that were injected with SARS-CoV became lethargic within 2 to 4 days postinfection, and one of the three ferrets died at day 4 postinfection (*1*,*2*). This finding indicates that domestic cats may not only be a useful animal model for evaluating vaccine and drugs candidates against SARS (*1*) but also be good reservoirs of SARS-CoV. Domestic cats living in the Amoy Gardens in Hong Kong, where >100 residents contracted SARS in the spring of 2003, were infected with SARS-CoV (*1*,*3*). This fact suggests that domestic cats can be naturally infected with SARS-CoV from humans infected with SARS, although how this SARS-CoV transmission occurs is unclear. More importantly, the frequency of the SARS-CoV strain that can be retransmitted from domestic animal to human, despite the widely accepted hypothesis of the animal origin of SARS-CoV (*4*–*6*), cannot be ascertained. If the transmission of SARS-CoV from animal to human is as easy as that from humans to domestic cats, the speculation that the outbreak of SARS in the Amoy Garden in Hong Kong was caused by environmental sources, such as U-traps in bathrooms contaminated with SARS-CoV, should be reevaluated (*3*). This outbreak of SARS in these apartments might also be caused by infected cats or other mammalian hosts.
